# ALDH1A2 Is a Candidate Tumor Suppressor Gene in Ovarian Cancer

**DOI:** 10.3390/cancers11101553

**Published:** 2019-10-14

**Authors:** Jung-A Choi, Hyunja Kwon, Hanbyoul Cho, Joon-Yong Chung, Stephen M. Hewitt, Jae-Hoon Kim

**Affiliations:** 1 Department of Obstetrics and Gynecology, Gangnam Severance Hospital, Yonsei University College of Medicine, Seoul 03722, Korea; jachoi@yuhs.ac (J.-A.C.); HJKWON89@yuhs.ac (H.K.); jaehoonkim@yuhs.ac (J.-H.K.); 2 Experimental Pathology Laboratory, Laboratory of Pathology, Center for Cancer Research, National Cancer Institute, National Institutes of Health, Bethesda, MD 20892, USA; chungjo@mail.nih.gov (J.-Y.C.); hewitts@mail.nih.gov (S.M.H.)

**Keywords:** ALDH1A2, methylation, DNMT, OVARIAN cancer, invasion

## Abstract

Aldehyde dehydrogenase 1 family member A2 (ALDH1A2) is a rate-limiting enzyme involved in cellular retinoic acid synthesis. However, its functional role in ovarian cancer remains elusive. Here, we found that ALDH1A2 was the most prominently downregulated gene among ALDH family members in ovarian cancer cells, according to complementary DNA microarray data. Low ALDH1A2 expression was associated with unfavorable prognosis and shorter disease-free and overall survival for ovarian cancer patients. Notably, hypermethylation of ALDH1A2 was significantly higher in ovarian cancer cell lines when compared to that in immortalized human ovarian surface epithelial cell lines. ALDH1A2 expression was restored in various ovarian cancer cell lines after treatment with the DNA methylation inhibitor 5-aza-2′-deoxycytidine. Furthermore, silencing DNA methyltransferase 1 (DNMT1) or 3B (DNMT3B) restored ALDH1A2 expression in ovarian cancer cell lines. Functional studies revealed that forced ALDH1A2 expression significantly impaired the proliferation of ovarian cancer cells and their invasive activity. To the best of our knowledge, this is the first study to show that ALDH1A2 expression is regulated by the epigenetic regulation of DNMTs, and subsequently that it might act as a tumor suppressor in ovarian cancer, further suggesting that enhancing ALDH1A2-linked signaling might provide new opportunities for therapeutic intervention in ovarian cancer.

## 1. Introduction

Ovarian cancer has the highest mortality rate among all gynecological cancers because most cases are not diagnosed until the disease has reached an advanced stage [1]. Although the five-year survival rate for ovarian cancer patients has substantially improved, owing to ongoing efforts to develop an effective screening strategy [1], the lack of reliable methods for early diagnosis and the absence of specific symptoms result in diagnoses at advanced stages [1]. Furthermore, approximately 80% of women with advanced ovarian cancer show tumor progression or, more commonly, recurrence [2]. Thus, understanding the pathogenesis of ovarian cancer and the molecular mechanism of its early stage is crucial for the management of this lethal, highly metastatic disease.

The aldehyde dehydrogenase 1 (ALDH1) family comprises major enzymes that produce retinoic acid (RA) via the oxidation of all-*trans* retinal and 9-*cis*-retinal, which is mainly involved in biological functions related to cell differentiation, cell cycle arrest, and eventually, apoptosis [3,4,5]. The ALDH1A subfamily consists of three members, ALDH1A1, ALDH1A2, and ALDH1A3 [6]. ALDH1A1 participates in retinal oxidation, acetaldehyde metabolism, and detoxification of cyclophosphamide, whereas the ALDH1A2 and ALDH1A3 proteins are integral to the oxidation of retinal to RA [7]. Interestingly, despite similarities in the structure and function of the isoenzymes of the ALDH1A subfamily [8], several lines of evidence suggest that these molecules might perform different functions in cancer progression [7,8,9,10,11,12,13,14]. ALDH1A1 has been implicated in conferring resistance to certain anticancer agents, such as cyclophosphamide, and was shown to play a functional role in cancer stem cells [11,12]. In contrast to that of ALDH1A1, low ALDH1A2 expression has been associated with an unfavorable prognosis in prostate cancer and head and neck squamous cell carcinoma patients [13,14]. Meanwhile, ALDH1A3 plays important roles in several metabolic and physiological processes [15], which potentially suggest different roles for ALDH1A isoforms in cancer progression. However, the functions and prognostic value of ALDH1A2 in ovarian cancer progression remain unclear.

The methylation of CpG sites is an epigenetic mechanism underlying the regulation of gene expression, which usually leads to gene silencing [16]. Aberrant methylation of gene promoter regions is one of the earliest molecular alterations that occurs during carcinogenesis and a key mechanism for the inactivation of tumor suppressor genes [16,17,18]. Therefore, methylation has emerged as a promising marker for the early detection of cancer [19]. In fact, CpG islands in tumor suppressor genes such as CDKN2B, BRACA1, RASSF1A, MLH1, RARB2, and TIMP3 undergo aberrant methylation in ovarian cancer [20]. DNA methylation patterns have been shown to be regulated by DNA methyltransferases (DNMTs), including DNMT1, DNMT3A, and DNMT3B [21]. DNMT1 is essential for maintaining DNA methylation patterns in proliferating cells [22], whereas DNMT3A and DNMT3B are required for *de novo* methylation during embryonic development [23]. However, there are no reports regarding the epigenetic regulation of *ALDH1A2* by DNMTs in human ovarian cancer.

Therefore, in this study, we evaluated the expression profiles of ALDH isozyme-encoding genes in ovarian cancer using a complementary DNA (cDNA) microarray. Additionally, we focused on the role of ALDH1A2 as one of the most prominently downregulated genes in ovarian cancer.

## 2. Results

### 2.1. Reduced Expression of ALDH1A2 in Ovarian Cancer

**Figure 1:**
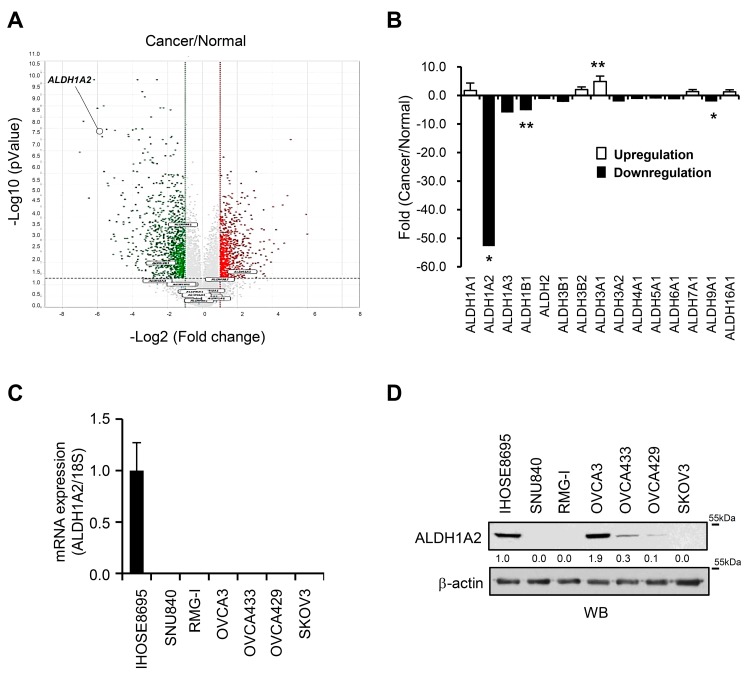
Comparative analysis of ALDH gene expression profiles and ALDH1A2 protein expression in ovarian cancer cell lines. (**A**) Volcano plot of the distribution of differentially expressed genes between ovarian cancer cell lines (YDOV-139, YDOV-157, YDOV-161, YDOV-13, YDOV-105, and YDOV-151) and human ovarian surface epithelial (HOSE) cell lines (HOSE 198, 209, 211, and 213) using a DNA microarray. The volcano plot shows that 24,957 genes were differentially expressed (*p* < 0.01 and fold change > 2). The −log10 (*p*-value) of each gene is plotted against the log 2 ratio of cancer intensity to normal intensity. Vertical dotted lines in red and green correspond to a 2.0-fold upregulation and 2.0-fold downregulation of expression, respectively. Horizontal black dotted lines indicate the significance level at *p* = 0.01. Plots were generated using ExDEGA v.1.6.5 software (Ebiogen, Seoul, Republic of Korea). (**B**) Up- and downregulation of gene expression were examined in six ovarian cancer cell lines (YDOV-139, YDOV-157, YDOV-161, YDOV-13, YDOV-105, and YDOV-151) and four HOSE cell lines (HOSE 198, 209, 211, and 213) using a DNA microarray. The values indicate fold changes in the expression levels of each gene in the ovarian cancer cell lines relative to those in the HOSE cell lines. Data are expressed as the means  ± SD. Statistical significance was assessed using an unpaired *t*-test. * *p* < 0.001, ** *p* < 0.05. (**C**) Real-time PCR of the cDNA microarray results of ALDH1A2 in ovarian cancer cell lines and immortalized HOSE cells. The 18S rRNA gene was used as the endogenous control to normalize the data. (**D**) Immunoblotting analysis of ALDH1A2 expression in ovarian cancer cell lines and immortalized HOSE cells, with β-actin used as a loading control. The changes in the protein levels were estimated by quantification of band intensities with the ImageJ software. The protein levels were normalized to the signal of β-actin.

To evaluate whether ALDH isozymes contribute to the progression of ovarian cancer, an Illumina microarray was used to identify differentially expressed ALDH genes in six ovarian cancer cell lines (YDOV-139, YDOV-157, YDOV-161, YDOV-13, YDOV-105, and YDOV-151) and four human ovarian surface epithelial (HOSE) cell lines (HOSE 198, 209, 211, and 213) [24]. RNA isolated from the cells was directly compared by hybridization with cDNA microarrays with 24,957 probe sets. The volcano plot shows that 24,957 genes were differentially expressed (*p* < 0.01 and fold change > 2) in the cancer cells compared to the normal cells (Figure 1A). We found that 15 ALDH isoforms exhibited differential expression patterns in the ovarian cancer cell lines compared to the normal cells (Figure 1A,B, Table S1). ALDH1A2, ALDH1B1, and ALDH9A1 were downregulated in the ovarian cancer cells compared to the expression in HOSE cells (Figure 1A and Table S1), whereas ALDH3A1 was upregulated in the ovarian cancer cells. Among these isoforms, we further characterized the functional role of ALDH1A2 in ovarian cancer progression, because it was one of the most prominently downregulated genes, with approximately 50-fold lower expression levels in ovarian cancer cells than in normal cells (Figure 1B, Table S1). These decreased expression levels were further validated by real-time PCR, and immunoblotting in ovarian cancer cell lines (SNU840, RMG1, OVCA433, OVCA429, and SKOV3) and IHOSE8695 cells (Figure 1C,D). Taken together, these observations suggest that the expression of ALDH1A2 is strongly downregulated in ovarian cancer.

### 2.2. ALDH1A2 Is Hypermethylated in Ovarian Cancer

**Figure 2:**
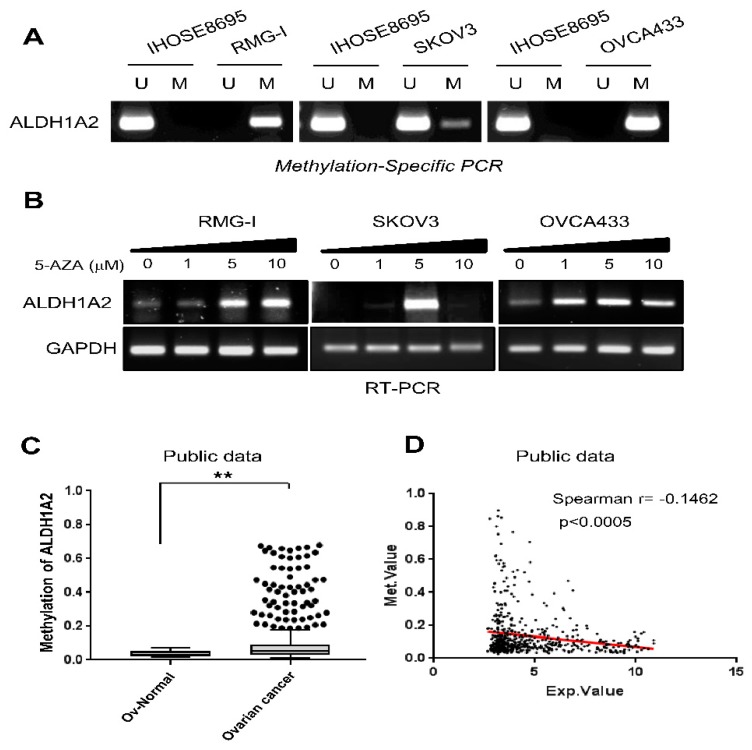
Methylation status of the ALDH1A2 gene in ovarian cancer. (**A**) Analysis of ALDH1A2 methylation in RMG1, SKOV3, and OVCA433 cells by methylation-specific PCR. U, primer pair specific to unmethylated DNA; M, primer pair specific to methylated DNA. (**B**) qRT-PCR analysis of ALDH1A2 mRNA levels in 5-Aza-CdR-treated and control cells. *GAPDH* was used as loading control. (**C**) Methylation status of ALDH1A2 in the Methylation and Expression database of Normal and Tumor tissues (MENT; http://mgrc.kribb.re.kr:8080/MENT/). A Mann–Whitney test was used to evaluate statistical significances. ** *p* < 0.05. (**D**) Correlation between DNA methylation and gene expression of ALDH1A2 were determined by Spearman’s correlation through the MENT database with clinical data with the TCGA ov dataset.

Methylation of CpG islands in gene promoter regions is known to be a mechanism underlying the inactivation of tumor suppressor genes, which is associated with the aberrant silencing of their transcription [25]. Thus, we first analyzed the DNA methylation status of the ALDH1A2 gene in ovarian cancer cell lines, RMG1, SKOV3, and OVCA433, which express low ALDH1A2 relative to levels in IHOSE8695, using conventional methylation-specific PCR analysis (MSP). Methylation-specific PCR analysis revealed that the methylation of ALDH1A2 was markedly higher in ovarian cancer cell lines (RMG1, SKOV3, and OVCA433) than in IHOSE8695 cells (Figure 2A). qRT-PCR demonstrated that the low levels of ALDH1A2 expression in ovarian cancer cell lines were reversed following treatment with the demethylation agent 5-Aza-CdR (Figure 2B). Next, we further confirmed the methylation status of the ALDH1A2 gene in a public dataset, Methylation and Expression database of Normal and Tumor tissues (MENT), and found that ALDH1A2 was significantly hypermethylated in ovarian cancer tissues compared to that in normal ovarian tissues (Figure 2C). In addition, correlation analysis of methylation and gene expression levels revealed a negative correlation (Spearman’s r = −0.1462, *p* < 0.0005) between DNA methylation and the gene expression of ALDH1A2 (Figure 2D). Taken together, these data suggest that ALDH1A2 expression is downregulated via gene methylation in ovarian cancer.

### 2.3. DNMTs Are Negative Upstream Regulators of ALDH1A2 Expression

**Figure 3:**
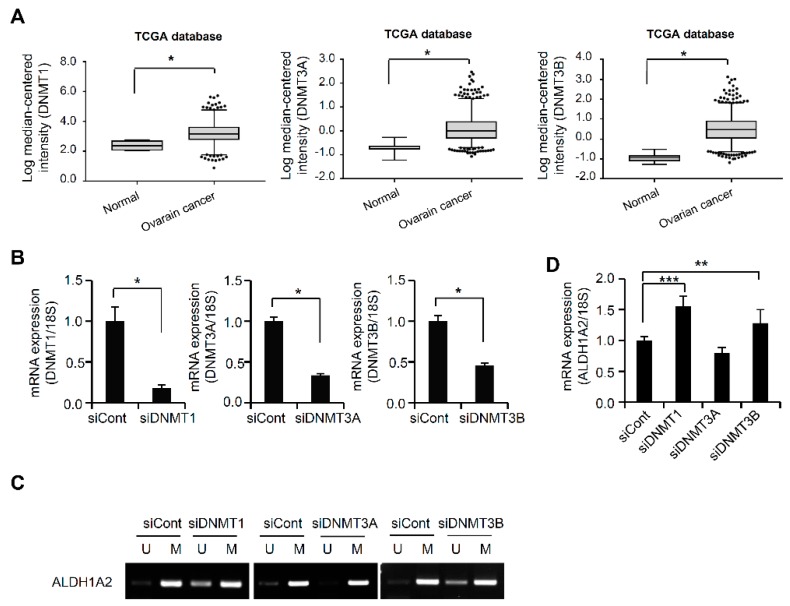
DNA methyltransferase (DNMT) is an upstream regulator of ALDH1A2 expression. (**A**) Data on the mRNA expression of DNMT1, DNMT3A, and DNMT3B obtained from the Oncomine database (http://www.oncomine.com). A Mann–Whitney test was used to evaluate statistical significances. * *p* < 0.001. (**B**) Expression of DNMT1, DNMT3A, and DNMT3B in RMG1 cells, as determined by real-time PCR. Knockdown of DNMT1, DNMT3A, and DNMT3B was performed by transfecting RMG1 cells with specific siRNAs, siDNMT1, siDNMT3A, and siDNMT3B, respectively. Nontargeting siRNA (siCont) was used as a control. The 18S rRNA gene was used as the endogenous control to normalize the data. Data are expressed as the means ± SD. Statistical significance was assessed using an unpaired *t*-test. * *p* < 0.001. (**C**) Analysis of ALDH1A2 methylation by methylation-specific PCR after knockdown of DNMT1, DNMT3A, and DNMT3B in RMG1 cells. Knockdown of DNMT1, DNMT3A, and DNMT3B was performed by transfecting RMG1 cells with specific siRNAs, siDNMT1, siDNMT3A, and siDNMT3B, respectively. The lysates were subjected to methylation-specific PCR. Nontargeting siRNA (siCont) was used as a control. U, primer pair specific to unmethylated DNA; M, primer pair specific to methylated DNA. (**D**) ALDH1A2 expression after transfection of RMG1 cells with siDNMT1, siDNMT3A, or siDNMT3B, as determined by real-time PCR. Nontargeting siRNA (siCont) was used as a control. The 18S rRNA gene was used as the endogenous control to normalize the real-time PCR data. ** *p* < 0.05, *** *p* < 0.005.

Since the DNMT family plays a pivotal role in maintaining DNA methylation patterns [21], we monitored the expression of different DNMT genes in ovarian cancer patients through the publicly available database Oncomine. The mRNA levels of DNMT1, DNMT3A, and DNMT3B were strongly elevated in ovarian cancer tissues compared to those in normal ovarian tissues, according to Oncomine (Figure 3A). To determine whether the silencing of DNMT in ovarian cancer cells can rescue the DNA hypermethylation of ALDH1A2, RMG1 cells were transfected with siRNA specific for the different DNMT genes (DNMT1, DNMT3A, and DNMT3B). The silencing of different DNMT genes by siRNA was confirmed by real-time PCR (Figure 3B). siRNA silencing of DNMT1 or DNMT3B restored the expression of ALDH1A2 and reduced methylation in ovarian cancer cells, whereas no significant changes in ALDH1A2 expression were observed upon knockdown of DNMT3A (Figure 3C,D). These results demonstrated that DNMT1 and DNMT3B contribute to the hypermethylation of ALDH1A2, suggesting that they negatively regulate ALDH1A2 expression in ovarian cancer.

### 2.4. ALDH1A2 Overexpression Attenuates the Proliferation and Invasion Potential of Ovarian Cancer Cell Lines

**Figure 4:**
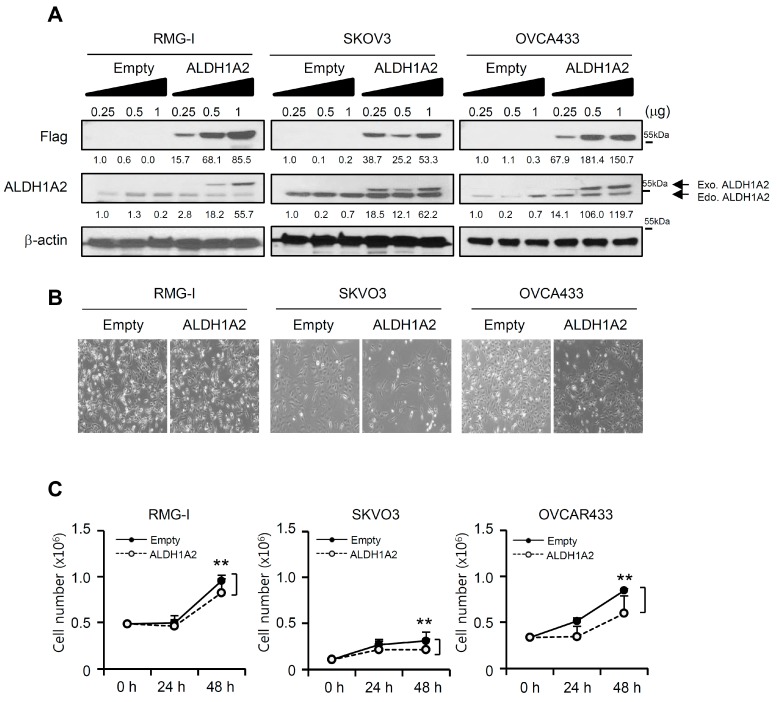
Effects of forced expression of ALDH1A2 on the proliferation of ovarian cancer cells. (**A**) Cells were transfected with vector expressing Flag-tagged ALDH1A2 or empty vector. After 24 h, whole lysates were immunoblotted with anti-ALDH1A2 and anti-Flag antibodies. β-Actin was used as a loading control. Densitometric analysis was performed by ImageJ software. The protein levels were normalized to the signal of β-actin. (**B**,**C**) Effects of forced expression of ALDH1A2 on the proliferation of ovarian cancer cell lines. Cells were transfected with Flag-tagged ALDH1A2 or empty vector. After indicated times, phase contrast images were observed (**B**). The cells were trypsinized with trypsin–EDTA, and the number of viable cells (**C**) was determined by a Vi-Cell XR cell viability analyzer at indicated time points. Original magnification: 20×. Statistical significance was assessed using an unpaired *t*-test. ** *p* < 0.05.

**Figure 5:**
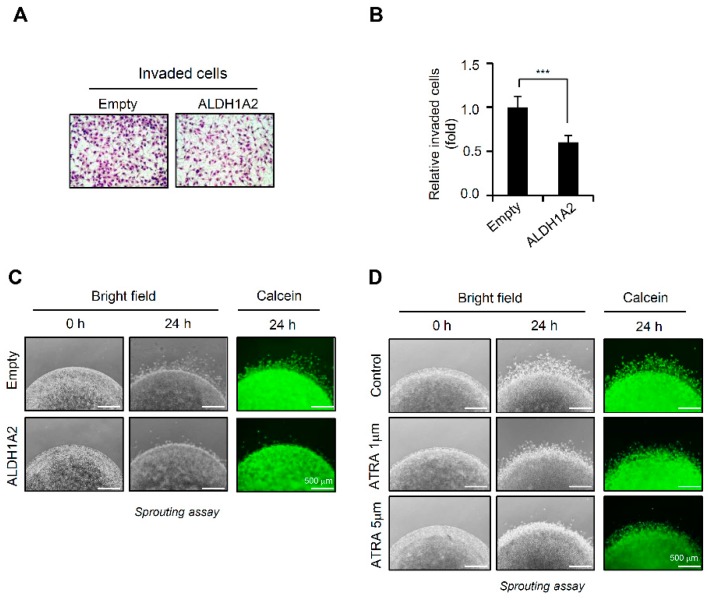
Effects of forced expression of ALDH1A2 on invasive potential of ovarian cancer cells. (**A**,**B**) Invasion assay; RMG-I cells transfected with either the empty vector or ALDH1A2 expression vector for 24 h were loaded into the upper chamber and then fetal bovine serum (FBS, 5%) was added to the lower chamber. After 24 h, the cells that invaded the lower chamber were stained with DiffQuik (**A**) and quantified (**B**) under a microscope. Data are expressed as means  ± SD. Statistical significance was assessed using an unpaired *t*-test. *** *p* < 0.005. (**C**) Sprouting assay; RMG1 cells ectopically expressing ALDH1A2 were embedded with Matrigel and incubated for 24 h. Phase contrast images were observed (left panel). Calcein-AM-stained cells were visualized using a fluorescence microscope (right panel). Original magnification: 4×. Scale bar, 500 μm. (**D**) Sprouting assay using RMG1 cells incubated in the presence or absence of ATRA (1 μM, 5 μM). Phase contrast images were observed (left panel). Calcein-AM-stained cells were visualized using a fluorescence microscope (right panel). Original magnification: 4×. Scale bar, 500 μm.

To elucidate the function of reduced ALDH1A2 in ovarian cancer progression, we constructed the pCMV-TagB-Flag-ALDH1A2 expression vector to overexpress ALDH1A2 in ovarian cancer cell lines. The overexpression of this construct in RMG1, SKOV3, and OVCA433 cells was confirmed by immunoblot analysis using antibodies against ALDH1A2 or Flag (Figure 4A) and by immunocytochemistry with anti-Flag antibodies (Figure S1). Forced expression of ALDH1A2 decreased cell proliferation in a time-dependent manner and induced S-phase cell cycle arrest (Figure 4B,C, Figure S2A), whereas it failed to affect cell death (Figures S2B and S3). Furthermore, the forced expression of ALDH1A2 significantly impaired the invasive behavior of RMG1 cells (Figure 5A,B). We further confirmed the effect of ALDH1A2 overexpression on the invasive capacity of cells by using Matrigel-embedded sprouting assays, which was evaluated as their ability to spread from 3D-matrigel spheroids (Figure 5C). The ALDH1A2 construct-transfected cells showed significantly lower spreading capacity than the empty vector-transfected cells. Next, as ALDH1A2 is the first enzyme to produce RA, we investigated the effect of ATRA, an active vitamin A metabolite, on inducing the constitutive activation of retinoid signaling. Treatment of ovarian cancer cell lines with ATRA significantly impaired their invasive capacity and spread from 3D Matrigel spheroids (Figure 5D). Collectively, these observations suggested that ALDH1A2 plays a critical role in mediating the invasion activity of ovarian cancer cells.

### 2.5. Downregulation of ALDH1A2 Correlates with Poor Prognosis in Ovarian Cancer Patients

**Figure 6:**
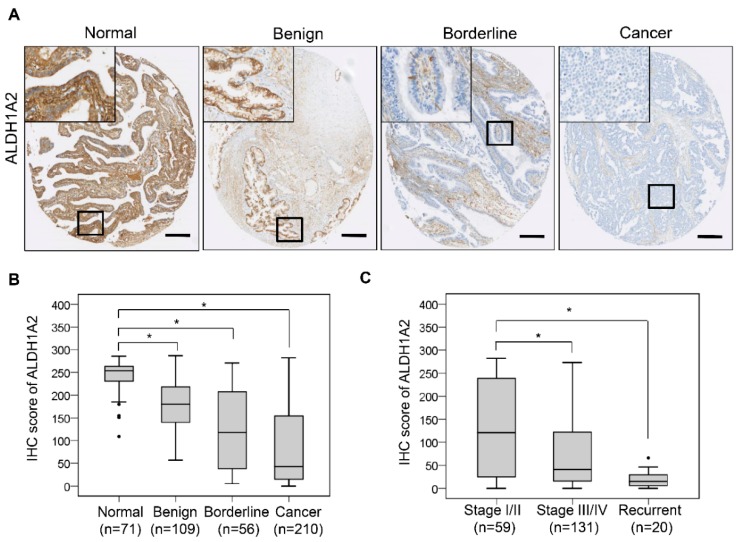
Reduced ALDH1A2 expression correlates with poor prognosis in ovarian cancer patients. (**A**) Representative images of immunohistochemical staining of ALDH1A2 expression in epithelial ovarian tissues from benign, borderline, and carcinoma patients and in nonadjacent normal tissues (Scale bar: 250 μm). (**B,C**) Box plot depicting the immunohistochemical staining data. Histoscores were computed based on the intensity and tissue area of positive staining. * *p* < 0.001.

To elucidate the clinical significance of ALDH1A2 in ovarian cancer patients, we compared ALDH1A2 expression levels by immunohistochemistry in benign, borderline, and malignant ovarian tumor tissues and in normal ovarian/fallopian epithelial tissues (Figure 6A). ALDH1A2 expression showed an obvious decrease with tumor progression from normal epithelium to ovarian cancer tissues (Figure 6B). The expression levels of ALDH1A2 are summarized in Table 1 according to clinical pathological characteristics of ovarian cancer patients. ALDH1A2 immunoreactivity was associated with an early tumor stage (*p* < 0.001) and serous cell type (*p* < 0.001; Figure 6C).

**Figure 7:**
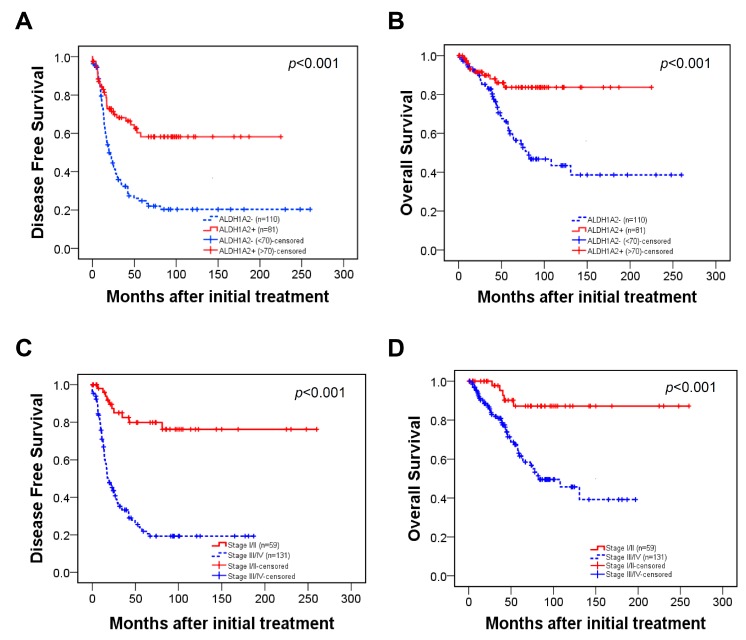
Kaplan–Meier survival curve for patients with epithelial ovarian cancer. (**A**,**B**) Patients with ALDH1A2^−^ tumors (histoscore < 70) showed significantly worse disease-free (**A**) and overall survival (**B**) than patients with ALDH1A2^+^ tumors. (**C,D**) Patients with advanced stages (III/IV) showed significantly worse disease-free (**C**) and overall survival (**D**) than patients with early-stage ovarian cancer.

We next examined the relationship between ALDH1A2 protein expression and patient survival outcomes. The Kaplan–Meier plots demonstrated that the patients with lower ALDH1A2 expression, as well as patients with advanced tumor stage (stage III/IV), displayed shorter disease-free (*p* < 0.001) and overall (*p* < 0.001) survival (Figure 7A–D). Multivariate analysis showed that ALDH1A2 expression (*p* = 0.012), FIGO stage (*p* < 0.001), and tumor grade (*p* = 0.032) were independent prognostic factors for disease-free survival (Table 2). The Cox proportional hazards model also showed that high ALDH1A2 expression (hazard ratio = 0.42, 95% confidence interval (CI) = 0.20–0.85, *p =* 0.016), high tumor stage (hazard ratio = 3.06, 95% CI = 1.18–7.93, *p =* 0.021), serous cell type (hazard ratio = 2.63, 95% CI = 1.03–6.73, *p* = 0.042), and old age (hazard ratio = 1.94, 95% CI = 1.06–3.53, *p =* 0.030) were independent prognostic factors of overall survival. Thus, these results show that ALDH1A2 expression is correlated with tumorigenesis, patient survival rate, and disease recurrence rate in ovarian cancer.

## 3. Discussion

In the present study, we identified ALDH1A2 as one of the most prominently downregulated ALDH genes in ovarian cancer cells, as compared to the expression in normal cells, based on microarray analysis. In addition, lower ALDH1A2 expression was correlated with poor prognosis in ovarian cancer patients. Furthermore, the ALDH1A2 gene was found to be hypermethylated via DNMT1 or DNMT3B in ovarian cancer cell lines, indicating that ALDH1A2 expression is regulated by epigenetic regulation via DNMT. The overexpression of ALDH1A2 also suppressed the proliferation and invasive ability of ovarian cancer cells. Taken together, our results suggest that ALDH1A2 might act as a tumor suppressor gene in ovarian cancer.

Indeed, compelling evidence has emerged in recent years, supporting a tumor-suppressive role of ALDH1A2. The expression of ALDH1A2 was shown to be reduced in cancer tissues compared to that in normal epithelial regions, which was associated with an unfavorable prognosis in prostate and head and neck cancers [13,14]. Furthermore, high ALDH1A2 transcription levels correlated with improved overall survival in breast cancer patients [26]. Seidensaal et al. [14] reported that inhibition of ALDH1A2 induces the loss of cell adhesion and a mesenchymal-like phenotype by regulating Twist, vimentin, and N-cadherin, thus indicating the possibility that low ALDH1A2 expression could be associated with an unfavorable prognosis. However, it had not been determined whether reduced ALDH1A2 is relevant to the early diagnosis of ovarian cancer. In these studies, we found that low ALDH1A2 expression gradually decreased with tumor progression from a normal epithelium to ovarian cancer tissues. The expression of ALDH1A2 at an advanced stage (stage III/IV) is lower than that at an early tumor stage (stage I/II). Furthermore, in stage I ovarian cancer (*n* = 44), CA125 was elevated in 28 (63.6%) of 44 patients. Of the 16 patients who presented false negative results for CA125, eight (50.0%) were ALDH1A2-negative (data not shown). Thus, these observations support our notion that ALDH1A2 is a potential biomarker contributing to early cancer detection, in conjunction with CA125, and prediction of prognosis. Therefore, these data led us to speculate that ALDH1A2 might be a tumor inhibitor in ovarian cancer.

Aberrant DNA methylation is widely considered one of the earliest molecular changes leading to the inactivation of tumor suppressor genes in carcinogenesis [16,17,18,19,20,21]. In ovarian cancer, aberrant methylation of CpG islands in tumor suppressor genes, including CDKN2B, BRCA1, APC, RASSF1, and CDH13, is a frequent event compared to that in the normal ovarian surface epithelium [27,28]. Tam et al. [29] observed that the frequency of methylation was the highest in ovarian cancer, followed by that in borderline and benign tissues, thus indicating that the detection of alterations in DNA methylation patterns has the potential to be applied for the detection of early-stage or, possibly, premalignant disease. Notably, methylation of circulating tumor DNA (ctDNA), which is tumor-derived fragmented DNA in the bloodstream, has been observed in ovarian cancer [30]. Methylation of genes such as RARB2, APC, CDKN2A, and RUNX3 has been shown to decrease following surgery in breast, esophageal, liver, and gastric cancers [31]. This suggests the possibility that monitoring the methylation of tumor-specific genes in blood could offer an alternative approach for tracking tumor response to therapy. However, to our knowledge, there has been no report regarding the epigenetic regulation of ALDH1A2 in human ovarian cancer. Here, we showed that a low level of ALDH1A2 expression in ovarian cancer was correlated with hypermethylation of the gene. Treatment of ovarian cancer cells with the demethylating agent 5-Aza-CdR reversed the repression of ALDH1A2 expression. Importantly, the hypermethylation of ALDH1A2 genes seems to be a common feature in various cancers such as brain tumors and prostate, colon, and kidney cancers, according to the MENT database (Figure S1). This suggests that ALDH1A2 is a novel hypermethylated gene in cancer. However, the detailed mechanism underlying the link between ALDH1A2 methylation and ovarian cancer progression requires further investigation. In the present study, although mRNA expression of ALDH1A2 in OVCA3 cells was low compared with that in normal cells, we failed to detect low levels of ALDH1A2 protein in OVCA3 cells. These results suggest the possibility that ALDH1A2 expression in OVCA3 cells may be regulated by post-transcriptional modifications of mRNA or action of miRNA. Due to the fact that the biological function of ALDH1A2 is exerted by the protein and not by the RNA, and OVCA3 cells display normal ALDH1A2 protein levels, this cell line represents an exception to the widespread ALDH1A2 downregulation in ovarian cancer.

DNMT are a conserved family of cytosine methylases with a key role in epigenetic regulation [32]. High expression of DNMT3A or DNMT3B has been observed in a large number of specimens from cancer patients, and increased DNMT3A expression is implicated in hepatocellular carcinogenesis [33]. Exosomal DNMT1 enhances the resistance to cisplatin in ovarian cancer cells [34]. Leu et al. [35] suggested that the downregulation of DNMT1 protein restores the expression of some inactive genes, such as RASSF1A and HIN-1, through a decrease in their methylation levels in an ovarian cancer cell line. Consistent with the data of these reports, we observed that the mRNA levels of DNMT1, DNMT3A, and DNMT3B were elevated in ovarian cancer tissues compared to those in normal epithelial ovarian cells. However, only the loss of DNMT1 or DNMT3B significantly restored the ALDH1A2 expression in ovarian cancer cell lines. These observations further support our notion that expression of ALDH1A2 is regulated by epigenetic regulation in ovarian cancer.

Although we observed the epigenetic regulation of ALDH1A2, the underlying mechanism of the correlation between low ALDH1A2 expression and aggressive disease in ovarian cancer remains unclear. A possible mechanism for the tumor suppressive effect of methylated ALDH1A2 can be mediated through RA signaling. Most biological effects of RA are mediated through retinoic acid receptors (RARα, RARβ, RARγ). RA treatment is known to induce the expression of RARβ, which functions as a tumor suppressor gene [36]. We observed that RARβ mRNA expression was low in ovarian cancer cell lines, relative to that in normal cells with our cDNA microarrays (Figure S5). Furthermore, we found that RARRES1, retinoic acid receptor responder 1, was strongly downregulated (approximately 14-fold) in ovarian cancer cells lines (Figure S5). The impaired mRNA levels of RARβ and RARRES1 in ovarian cancer tissues were further confirmed in the Oncomine database, suggesting that expression of retinoid-related genes is altered in human ovarian cancer cells (Figure S6). A recent study suggested that inhibition of ALDH1A2-RAR signaling induces loss of cell adhesion and gain of a mesenchymal-like phenotype [14]. Therefore, the methylation-silenced expression of ALDH1A2 may be responsible for lower RA levels and altered RA-linked signaling, contributing to the development of ovarian cancer progression. Another possible mechanism is via the PI3K/AKT/mTOR pathway, which is known as a potential predictor of the distinct invasive and migratory capacities of human ovarian cancer cells [37], but we failed to detect phosphorylation of AKT by ALDH1A2 overexpression in ovarian cancer cells (data not shown).

In summary, we obtained novel evidence showing that low expression levels of ALDH1A2 are correlated with an early tumor stage and poor prognosis for ovarian cancer patients, and that its expression is negatively regulated by the methylation of ALDH1A2 via DNMT1 or DNMT3B. Therefore, we suggest that DNA methylation of ALDH1A2 and the expression of ALDH1A2 might serve as reliable markers for the early diagnosis of disease to improve the outcome for patients with ovarian cancer, considering that an accurate and timely diagnosis is often crucial for treatment selection. These findings further raise the possibility that the methylation of ALDH1A2 could help to distinguish between relatively indolent and aggressive tumors, which can aid in decision making between more aggressive or less aggressive treatment and monitoring.

## 4. Materials and Methods

### 4.1. Cell Culture and Reagents

Human ovarian cancer cell lines, SKOV3, OVCA3, OVCA433, and OVCA429 were obtained from the American Type Culture Collection (Manassas, VA, USA). SNU-840 cells were purchased from Korea Cell Line Bank (KCLB, Seoul, Korea). RMG-I cells were purchased from Japanese Collection of Research Bioresources Cell Bank (JCRB, Tokyo, Japan). All purchased cell lines were cultured in Dulbecco’s modified Eagle’s medium (DMEM) supplemented with 10% fetal bovine serum (FBS; HyClone, UT, USA) and 1% antibiotics (Cellgro, Manassas, VA, USA). Six ovarian cancer cell lines (YDOV-139, YDOV-157, YDOV-161, YDOV-13, YDOV-105, and YDOV-151) and four HOSE cell lines (HOSE 198, 209, 211, and 213), which had been previously established [24], were maintained in a 1:1 mixture of Medium 199 and MCDB 105 (Sigma, St. Louis, MO, USA) supplemented with 10% FBS (Gemini Bio-Products, Calabasas, CA, USA). Immortalized HOSE8695 (IHOSE8695) cells had been established previously [38] and were maintained in DMEM supplemented with 10% FBS (HyClone, UT, USA) and 1% antibiotics (Cellgro, Manassas, VA, USA). All cell lines were incubated at 37 °C in a humidified incubator with 5% CO_2_. All-*trans* retinoic acid (ATRA) and 5-aza-2′-deoxycytidine (5-Aza-CdR) were purchased from Sigma (St. Louis, MO, USA).

### 4.2. Western Blot Analysis

For Western blot analysis, cells were harvested and lysed with ice-cold RIPA buffer (25 mM tris–HCl, pH 7.6, 150 mM NaCl, 1% NP-40, 1% sodium deoxycholate, and 0.1% sodium dodecyl sulfate) containing a protease inhibitor cocktail (Roche Applied Science, Mannheim, Germany). Proteins from cell lysates were resolved by 10% sodium dodecyl sulfate polyacrylamide gel electrophoresis and then transferred to a nitrocellulose membrane (Pierce, Rockford, IL, USA) using an electric transfer system. The membrane was incubated with the following antibodies: anti-ALDH1A2, anti-Flag (Santa Cruz Biotechnology, Santa Cruz, CA, USA), and anti-β-actin (Sigma–Aldrich) as a loading control. The membrane was then incubated with horseradish peroxidase-conjugated goat anti-mouse or anti-rabbit secondary antibodies, and immunoreactive bands were visualized using enhanced chemiluminescence reagents (Santa Cruz Biotechnology, Santa Cruz, CA, USA).

### 4.3. RNA Preparation and cDNA Microarray Hybridization

Total RNA was extracted and purified from six ovarian cancer cell lines (YDOV-139, YDOV-157, YDOV-161, YDOV-13, YDOV-105, and YDOV-151) and four human ovarian surface epithelial (HOSE) cell lines (HOSE 198, 209, 211, and 213) using the TRIzol reagent (Invitrogen Life Technologies, Carlsbad, CA, USA) and the RNeasy kit (Qiagen, Inc., Valencia, CA, USA) according to the manufacturers’ suggested protocols. Probes were prepared for each cell line and hybridized independently in the microarrays. Thus, the array comprised of 10 samples—6 tumor and 4 normal—that were appropriately grouped for differential expression analysis. Biotinylated cRNA was produced by T7 in vitro transcription using the Ambion Illumina RNA amplification kit (Ambion, Inc., Austin, TX, USA). We verified the quality of total RNA by gel analysis with the total RNA Nano chip assay on an Agilent 2100 bioanalyzer (Agilent Technologies GmbH, Berlin, Germany). We performed chip hybridization, washing, cyanine 3–streptavidin (GE Healthcare, Amersham Biosciences, Uppsala, Sweden) staining, and scanning on the Illumina Bead Station 500 platform (Illumina, Inc., San Diego, CA, USA) using reagents and protocols supplied by the manufacturer. Hybridization of 750 ng of labeled cRNA was performed with biological duplicates on a Sentrix Human Ref-6-V2 Expression Bead chip (Illumina, San Diego, CA, USA) Scanning of arrays was performed using an Illumina Bead Array reader confocal scanner (Illumina, San Diego, CA, USA) according to the manufacturer’s instructions. Our records have been assigned the following GEO accession number: GSE135508.

### 4.4. Quantitative Reverse Transcription–Polymerase Chain Reaction (qRT-PCR) and Real-Time PCR

Total RNA was extracted from cells using the TRIzol reagent (Invitrogen, Carlsbad, CA, USA), and 1.25 µg of the total RNA was used for cDNA synthesis with the Maxima first-strand cDNA synthesis kit (Thermo Fisher Scientific, Villebon-sur-Yvette, France) according to the manufacturer’s protocol. PCR products were amplified with the primers listed in Table S1. qRT-PCR was performed and the data were analyzed on a C1000 thermal cycler (Bio-Rad, Hercules, CA, USA) using the following program: 33 cycles of denaturation at 95 °C for 30 s, annealing at 58 °C (ALDH1A2) or 58 °C (glyceraldehyde 3-phosphate dehydrogenase, GAPDH) for 30 s, and elongation at 72 °C for 40 s. The PCR products were evaluated by 1.8% agarose gel electrophoresis, using the GAPDH gene transcript as a control, and visualized using a Gel Doc XR molecular imager system (Bio-Rad, Hercules, CA, USA).

Real-time PCR was performed using the TOPreal™ qPCR 2× premix (SYBR Green with high ROX; Enzynomics) on an Applied Biosystems 7300 real-time PCR system (Applied Biosystems, Darmstadt, Germany). The reaction conditions were as follows: preincubation at 94 °C for 10 min, followed by 40 cycles of 94 °C for 10 s, 60 °C for 15 s, and 72 °C for 15 s, and a melting curve program, with the temperature rising from 60 °C to 95 °C. Relative mRNA expression levels were calculated using the comparative cycle threshold (2^−ΔΔCt^) method, with the 18S rRNA gene used as the endogenous control to normalize the data.

### 4.5. Methylation-Specific PCR Analysis

Genomic DNA was isolated from cultured cells with the GeneJET Genomic DNA Purification Kit (Thermo Fisher Scientific Baltics UAB, and Vilnius, Lithuania). Bisulfite modification of genomic DNAs was performed using the EpiJET Bisulfite Conversion Kit (Thermo Fisher Scientific Baltics UAB, and Vilnius, Lithuania) according to the manufacturer’s protocol. ALDH1A2 is associated with a ~1.5 kb CpG island at its 5′ end, overlapping with the transcription start site [13]. For methylation-specific PCR analysis, we amplified a 200 bp region within the CpG island upstream of the ALDH1A2 transcription start site using primer pairs specific for either methylated or unmethylated bisulfite-modified sequences (Table S2), as previously reported [13]. PCR was performed on a C1000 thermal cycler instrument (Bio-Rad, Hercules, CA, USA) using 200 ng of a bisulfite-modified DNA template, 1× EpiTaq PCR buffer, 0.3 mM dNTPs, 2.5 mM MgCl_2_, 200 ng of each primer, and 1.25 U EpiTaq DNA polymerase (TaKaRa EpiTaq™ HS for bisulfite-treated DNA; Takara, Nagoya, Japan) in a 50 µL reaction for 40 cycles (94 °C, 30 s; 65 °C, 45 s; and 72 °C, 1 min), followed by a 1.8% agarose gel electrophoresis and visualization using a Gel Doc XR molecular imager system (Bio-Rad, Hercules, CA, USA).

### 4.6. Public Databases

DNA methylation analysis of the ALDH1A2 gene in normal and ovarian tumor tissues of ovarian cancer patients was performed using the Methylation and Expression database of Normal and Tumor tissues (MENT; http://mgrc.kribb.re.kr:8080/MENT/), together with clinical data from the Gene Expression Omnibus (GEO) database (GEO accession numbers: GSE26989, GSE28648, GSE31826, and ov) and The Cancer Genome Atlas (TCGA, ov dataset). Methylation values were calculated as the average beta value for measuring methylation levels at each CpG site, ranging from 0 (least methylated) to 1 (most methylated). No further normalization of the derived beta value was performed. We selected cg00930873A for CpG probes from the MENT database for the beta values. Despite the large number of cancer samples, the difference in the group size was too large to be a meaningful statistic for the beta value in ovarian cancer. Relative to cancer samples, the normal sample size was too small. Therefore, data manipulations for patient selection were considered. A percentile cutoff for the beta value was selected at the 95th percentile, and genes were sorted by their beta value at the selected cutoff. Sorting data are displayed as a box plot. Statistical analyses were performed with the one-tailed Mann-Whitney test using GraphPad Prism 7 because the data did not follow a normal distribution. Correlation analysis of DNA methylation and the gene expression of ALDH1A2 was performed using MENT with clinical data from the TCGA ov dataset. mRNA levels of DNMT1, DNMT3A, and DNMT3B in ovarian cancer patients were determined by analysis of the TCGA Ki dataset, which is available through Oncomine (Compendia Bioscience, https://www.oncomine.org).

### 4.7. ALDH1A2 Expression Constructs and Transfection

The full-length coding sequence of *ALDH1A2* was PCR-amplified from a cDNA of IHOSE8695 using gene-specific primers, AAGCCCGGGCGGATCCATGACTTCCAGCAAGATA (forward) and GCTTGATATCGAATTCTTAGGAGTTCTTCTGGGG (reverse). The sequence was cloned in-frame into the *Bam*HI and *Eco*RI sites of pCMV-Tag2B-Flag (Stratagene, La Jolla, CA, USA) and confirmed by DNA sequencing. Subsequently, cells were plated and transfected with pCMV-Tag2B-Flag-ALDH1A2 or the empty vector using the Lipofectamine 2000 reagent (Invitrogen) according to the manufacturer’s recommendations. Whole cell lysates were subjected to Western blotting analysis for the confirmation of ALDH1A2 expression.

### 4.8. Cell Proliferation Assay

Cells were plated at a density of 0.2 × 10^6^ cells per well in 6-well plates and cultured for 24 h. The cells were then transfected with the ALDH1A2 expression vector or empty vector for 24 h and incubated for the indicated times at 37 °C. After the incubation, the cells were trypsinized with trypsin–EDTA, and the number of viable cells was determined by a Vi-Cell XR cell viability analyzer (Beckman Coulter, Inc., Brea, CA, USA).

### 4.9. Invasion Assay

To monitor the invasive potential of ovarian cancer cells, an invasion assay was performed using a 10-well Chemotaxis Chamber (Neuro Probe, Inc., Gaithersburg, MD, USA) with a 8 μm polycarbonate filter (Neuro Probe, Inc., Gaithersburg, MD, USA) coated with rat tail collagen type I (BD Bioscience) according to the manufacturer’s instructions. Briefly, 2 × 10^4^ cells were resuspended in a serum-free medium (285 μL) and plated onto the upper chamber. As a chemoattractant, DMEM containing 5% FBS (400 μL) was added to the lower chamber, and the chamber was incubated at 37 °C in a humidified incubator with 5% CO_2_. After 24 h, the membranes were fixed with 100% methanol and stained with a DiffQuik stain kit (Triangle Biomedical Sciences, Inc., Durham, NC, USA). The uninvaded cells were removed from the upper surface of the membrane, and the invaded cells were counted in six random high-power fields per filter using an Axio Imager M2 microscope (Carl Zeiss, Thornwood, NY, USA). Each experiment was repeated three times.

### 4.10. Matrigel-Embedded Sprouting Assay

The sprouting assay was performed according to a previously described method [39]. Briefly, cells were spotted at a density of 1×10^6^ cells and embedded in a 1:1 mixture of DMEM and growth factor-reduced Matrigel in a 6-well plate. After polymerization at 37 °C, the cells were incubated with DMEM supplemented with 10% FBS for 24h. Then, cells were stained with calcein-AM (Invitrogen, Carlsbad, CA, USA) to visualize cells that spread from the spheroid. Randomly chosen fields (4×) in each well were photographed using a fluorescence microscope (EVOS FL; AMG).

### 4.11. Small Interfering RNA (siRNA) Transfection

siRNAs against DNMT1, DNMT3A, and DNMT3B and a negative control siRNA were purchased from Santa Cruz Biotechnology (Santa Cruz, CA, USA). Cells were seeded at 2 × 10^5^ cells per well in 6-well plates and transfected with siRNAs (10 nM) using the Lipofectamine RNAiMAX reagent (Invitrogen) according to the manufacturer’s instructions.

### 4.12. Tissue Specimens

A total of 210 ovarian cancer, 56 borderline, 109 benign, and 71 nonadjacent normal epithelial tissues were obtained from patients at the Gangnam Severance Hospital between 1996 and 2010. Some of the paraffin blocks were provided by the Korea Gynecologic Cancer Bank through the Bio and Medical Technology Development Program of the Ministry of the National Research Foundation (NRF), funded by the Korean government (MSIT) (NRF-2017M3A9B8069610). Tissue samples were collected from patients after they provided written informed consent, and the study was approved by the Regional Institutional Review Board of the Gangnam Severance Hospital (3-2018-0194). Ovarian cancer was staged according to the International Federation of Gynecology and Obstetrics (FIGO) classification and graded according to the World Health Organization grading system. Clinical and pathological records were reviewed to collect data including age, surgical procedure, survival time, survival status, tumor grade, and cell type. The response to therapy was assessed according to the Response Evaluation Criteria in Solid Tumors (RECIST; version 1.0) by spiral computed tomography [40].

### 4.13. Tissue Microarray Construction and Immunohistochemistry

Tissue microarrays were produced from archival formalin-fixed, paraffin-embedded tissue blocks, and representative areas were meticulously selected from hematoxylin and eosin-stained slides. Tissue cylinders of 1.0 mm in diameter were extracted from selected areas of donor blocks and transplanted into recipient blocks using a tissue arrayer (Beecher Instruments, Inc., Silver Spring, MD, USA). For immunohistochemical staining, the tissue microarray blocks were cut into serial 5 mm thick sections, which were deparaffinized in xylene and rehydrated through a graded alcohol series to distilled water. Heat-induced antigen retrieval was performed for 20 min in an antigen retrieval buffer, pH 6 (Dako, Carpinteria, CA, USA). Endogenous peroxidase activity was blocked by incubation with 3% H_2_O_2_ for 10 min. The sections were incubated with an anti-ALDH1A2 mouse monoclonal antibody (clone no. G-2; Santa Cruz Biotechnology) at a 1:200 dilution for 1 h. The antigen–antibody reaction was detected with EnVision+ Dual Link System-HRP (Dako) and visualized with 3,3′-diaminobenzidine (Dako). The stained sections were lightly counterstained with hematoxylin. Appropriate negative and positive controls were concurrently analyzed.

Immunohistochemically stained slides were scanned by a NanoZoomer 2.0 HT (Hamamatsu Photonics K.K., Hamamatsu City, Japan) with a 20× objective magnification (0.5 µm resolution). The captured digital images were analyzed using the Visiopharm software version 4.5.1.324 (Visiopharm, Hørsholm, Denmark). The intensity of brown staining (0 = negative, 1 = weak, 2 = moderate, and 3 = strong) was obtained using a predefined algorithm and optimized settings. The overall immunohistochemical score was calculated by multiplying the intensity and percentage of staining, resulting in a score of 0 to 300. The Mann–Whitney *U*-test or Kruskal–Wallis test was used to compare the protein expression levels between groups. For survival analysis, expression values were dichotomized (positive vs. negative), with cutoff values showing the most discriminative power (histoscore of 70 for ALDH1A2). Survival distributions were estimated using the Kaplan–Meier method with a log-rank test. A Cox multivariate proportional hazards model was used to identify independent predictors of survival. Statistical analysis was performed using SPSS version 23.0 (SPSS, Chicago, IL, USA). In all cases, *p* < 0.05 was considered statistically significant.

### 4.14. Statistical Analysis

Results are expressed as the mean ± SD. based on the control. Statistical analyses were performed with Student’s *t*-test, Mann–Whitney test, or Spearman’s rank correlation coefficient using GraphPad Prism 7 (GraphPad Software, Inc., La Jolla, CA, USA). A *p*-value < 0.05 was considered as significant.

## Figures and Tables

**Table 1:** Expression of ALDH1A2 in relation to clinicopathological characteristics of ovarian cancer patients.

Characteristics	No.	%	Mean Score (95% CI)	Range	*p*-Value
All study subjects	446	100	136.4 (127.7–145.1)	0–287	
Diagnostic category					<0.001
Normal	71	15.9	240.8 (232.7–248.9)	109–286	
Benign	109	24.4	174.0 (164.2–183.8)	57–287	
Borderline	56	12.6	127.1 (103.9–150.4)	5–271	
Cancer	210	47.1	84.2 (72.1–96.1)	0–282	
FIGO stage					<0.001
I–II	59	28.1	126.4 (99.6–153.1)	0–282	
III–IV	131	61.9	75.5 (61.8–89.2)	0–273	
Recurrent	20	10.0	18.9 (11.2–26.6)	0–66	
Cell type					<0.001
Serous	144	68.6	67.9 (54.9–81.0)	0–279	
Others	66	31.4	119.4 (95.7–143.2)	0–282	
Tumor grade					0.142
Well/Moderate	87	45.3	93.2 (73.6–112.8)	0–282	
Poor	105	54.7	74.6 (58.6–90.6)	0–279	
CA125					0.598
Negative	34	16.4	92.5 (58.3–126.6)	0–282	
Positive (>35 U/mL)	173	83.6	83.7 (70.7–96.7)	0–280	
Platinum sensitivity					0.984
Sensitive	174	90.6	81.8 (68.8–94.8)	0–280	
Resistant	18	9.4	82.2 (34.0–130.5)	6–270	

FIGO, International Federation of Gynecology and Obstetrics; CA125, cancer antigen 125; CI, confidence interval.

**Table 2:** Univariate and multivariate analysis of the associations between prognostic variables and disease-free and overall survival in epithelial ovarian cancer patients.

Variable	Disease-Free Survival Hazard Ratio (95% CI), *p*-Value		Overall Survival Hazard Ratio (95% CI), *p*-Value	
	Univariate	Multivariate	Univariate	Multivariate
FIGO stage (III–IV)	6.40 (3.31–12.34), <0.001	3.81 (1.84–7.86), <0.001	5.09 (2.02–12.86), 0.001	3.06 (1.18–7.93), 0.021
Cell type (serous)	3.03 (1.80–5.11), <0.001	1.69 (0.92–3.09), 0.087	4.52 (1.79–11.38), 0.001	2.63 (1.03–6.73), 0.042
Tumor grade (poor)	1.95 (1.28–2.96), 0.002	1.60 (1.04–2.46), 0.032	1.68 (0.94–3.00), 0.076	NA
CA125^+^ (>35 U/mL)	2.38 (1.20–4.73), 0.013	1.05 (0.49–2.27), 0.882	2.21 (0.79–6.16), 0.127	NA
Age (>50 years)	1.57 (1.06–2.34), 0.024	1.28 (0.84–1.95), 0.247	2.12 (1.17–3.84), 0.013	1.94 (1.06–3.53) 0.030
ALDH1A2^+^	0.37 (0.24–0.58), <0.001	0.54 (0.33–0.87), 0.012	0.30 (0.15–0.60), 0.001	0.42 (0.20–0.85), 0.016

FIGO, International Federation of Gynecology and Obstetrics; CA125, cancer antigen 125; CI, confidence interval; NA, not applicable.

## Supplementary Materials

The following are available online at https://www.mdpi.com/2072-6694/11/10/1553/s1, Figure S1: Transfection efficiency after pCMV-tagB-Flag-ALDH1A2 transfection in RMG-I, SKOV3, and OVCA433 cells; Figure S2: Effect of ALDH1A2 overexpression on cell cycle distribution and cell death in ovarian cancer; Figure S3. Annexin V/PI staining in RMG-I, SKOV3, and OVCA433 cells overexpressing ALDH1A2.; Figure S4: Methylation status of *ALDH1A2* genes in public datasets, Methylation and Expression Database of Normal and Tumor Tissues (MENT; http://mgrc.kribb.re.kr:8080/MENT/); Figure S5: Expression of retinoic acid-related genes in ovarian cancer cell lines.; Figure S6: Data on the mRNA expression of *RARβ* and *RARRES1* obtained from the Oncomine database (http://www.oncomine.com).; Figure S7: Original blots for Western blot analyses shown in Figure 1D; Figure S8: Original blots for Western blot analyses shown in Figure 4A. Table S1: Up- and downregulated *ALDH* genes in ovarian cancer cells; Table S2: Primer sequences for qRT-PCR and real-time PCR; Table S3: Primer sequences for methylation-specific PCR.

## References

[1] Bast R.C., Hennessy B., Mills G.B. (2009). The biology of ovarian cancer: New opportunities for translation. Nat. Rev. Cancer.

[2] Luvero D., Milani A., Ledermann J.A. (2014). Treatment options in recurrent ovarian cancer: Latest evidence and clinical potential. Ther. Adv. Med. Oncol..

[3] Gudas L.J. (2012). Emerging roles for retinoids in regeneration and differentiation in normal and disease states. Biochim. Biophys. Acta.

[4] Bushue N., Wan Y.J. (2010). Retinoid pathway and cancer therapeutics. Adv. Drug Deliv. Rev..

[5] Tang X.H., Gudas L.J. (2011). Retinoids, retinoic acid receptors, and cancer. Annu. Rev. Pathol..

[6] Marcato P., Dean C.A., Giacomantonio C.A., Lee P.W. (2011). Aldehyde dehydrogenase: Its role as a cancer stem cell marker comes down to the specific isoform. Cell Cycle.

[7] Vasiliou V., Nebert D.W. (2005). Analysis and update of the human aldehyde dehydrogenase (ALDH) gene family. Hum. Genom..

[8] Morgan C.A., Hurley T.D. (2015). Characterization of Two Distinct Structural Classes of Selective Aldehyde Dehydrogenase 1A1 Inhibitors. J. Med. Chem..

[9] Chute J.P., Muramoto G.G., Whitesides J., Colvin M., Safi R., Chao N.J., McDonnell D.P. (2006). Inhibition of aldehyde dehydrogenase and retinoid signaling induces the expansion of human hematopoietic stem cells. Proc. Natl. Acad. Sci. USA.

[10] Corti S., Locatelli F., Papadimitriou D., Donadoni C., Del Bo R., Crimi M., Bordoni A., Fortunato F., Strazzer S., Menozzi G. (2006). Transplanted ALDHhiSSClo neural stem cells generate motor neurons and delay disease progression of nmd mice, an animal model of SMARD1. Hum. Mol. Genet..

[11] Condello S., Morgan C.A., Nagdas S., Cao L., Turek J., Hurley T.D., Matei D. (2015). β-Catenin-regulated ALDH1A1 is a target in ovarian cancer spheroids. Oncogene.

[12] Moreb J.S., Mohuczy D., Ostmark B., Zucali J.R. (2007). RNAi-mediated knockdown of aldehyde dehydrogenase class-1A1 and class-3A1 is specific and reveals that each contributes equally to the resistance against 4-hydroperoxycyclophosphamide. Cancer Chemother. Pharmacol..

[13] Kim H., Lapointe J., Kaygusuz G., Ong D.E., Li C., van de Rijn M., Brooks J.D., Pollack J.R. (2005). The retinoic acid synthesis gene ALDH1a2 is a candidate tumor suppressor in prostate cancer. Cancer Res..

[14] Seidensaal K., Nollert A., Feige A.H., Muller M., Fleming T., Gunkel N., Zaoui K., Grabe N., Weichert W., Weber K.J. (2015). Impaired aldehyde dehydrogenase 1 subfamily member 2A-dependent retinoic acid signaling is related with a mesenchymal-like phenotype and an unfavorable prognosis of head and neck squamous cell carcinoma. Mol. Cancer.

[15] Duan J.J., Cai J., Guo Y.F., Bian X.W., Yu S.C. (2016). ALDH1A3, a metabolic target for cancer diagnosis and therapy. Int. J. Cancer.

[16] Lund A.H., van Lohuizen M. (2004). Epigenetics and cancer. Genes Dev..

[17] Laird P.W. (2005). Cancer epigenetics. Hum. Mol. Genet..

[18] Ducasse M., Brown M.A. (2006). Epigenetic aberrations and cancer. Mol. Cancer.

[19] Paluszczak J., Baer-Dubowska W. (2006). Epigenetic diagnostics of cancer--the application of DNA methylation markers. J. Appl. Genet..

[20] Ozdemir F., Altinisik J., Karateke A., Coksuer H., Buyru N. (2012). Methylation of tumor suppressor genes in ovarian cancer. Exp. Ther. Med..

[21] Lyko F. (2018). The DNA methyltransferase family: A versatile toolkit for epigenetic regulation. Nat. Rev. Genet..

[22] Spada F., Haemmer A., Kuch D., Rothbauer U., Schermelleh L., Kremmer E., Carell T., Längst G., Leonhardt H. (2007). DNMT1 but not its interaction with the replication machinery is required for maintenance of DNA methylation in human cells. J. Cell Biol..

[23] Okano M., Bell D.W., Haber D.A., Li E. (1999). DNA Methyltransferases Dnmt3a and Dnmt3b Are Essential for De Novo Methylation and Mammalian Development. Cell.

[24] Cho H., Kim J.H. (2009). Lipocalin2 expressions correlate significantly with tumor differentiation in epithelial ovarian cancer. J. Histochem. Cytochem..

[25] Herman J.G., Baylin S.B. (2003). Gene silencing in cancer in association with promoter hypermethylation. N. Engl. J. Med..

[26] Wu S., Xue W., Huang X., Yu X., Luo M., Huang Y., Liu Y., Bi Z., Qiu X., Bai S. (2015). Distinct prognostic values of ALDH1 isoenzymes in breast cancer. Tumour Biol..

[27] Balch C., Fang F., Matei D.E., Huang T.H., Nephew K.P. (2009). Minireview: Epigenetic changes in ovarian cancer. Endocrinology.

[28] Asadollahi R., Hyde C.A., Zhong X.Y. (2010). Epigenetics of ovarian cancer: From the lab to the clinic. Gynecol. Oncol..

[29] Tam K.F., Liu V.W., Liu S.S., Tsang P.C., Cheung A.N., Yip A.M., Ngan H.Y. (2007). Methylation profile in benign, borderline and malignant ovarian tumors. J. Cancer Res. Clin. Oncol..

[30] Eichner J., Rujan T., Yang Z., Teschendorff A.E., Ryan A., Cibula D., Menon U., Wittenberger T. (2017). The potential of circulating tumor DNA methylation analysis for the early detection and management of ovarian cancer. Genome Med..

[31] Warton K., Mahon K.L., Samimi G. (2016). Methylated circulating tumor DNA in blood: Power in cancer prognosis and response. Endocrine-Related Cancer.

[32] Easwaran H., Tsai H.C., Baylin S.B. (2014). Cancer epigenetics: Tumor heterogeneity, plasticity of stem-like states, and drug resistance. Mol. Cell.

[33] Zhao Z., Wu Q., Cheng J., Qiu X., Zhang J., Fan H. (2010). Depletion of DNMT3A suppressed cell proliferation and restored PTEN in hepatocellular carcinoma cell. J. Biomed. Biotechnol..

[34] Cao Y.L., Zhuang T., Xing B.H., Li N., Li Q. (2017). Exosomal DNMT1 mediates cisplatin resistance in ovarian cancer. Cell Biochem. Funct..

[35] Leu Y.W., Rahmatpanah F., Shi H., Wei S.H., Liu J.C., Yan P.S., Huang T.H. (2003). Double RNA interference of DNMT3b and DNMT1 enhances DNA demethylation and gene reactivation. Cancer Res..

[36] Sun S.Y., Wan H., Yue P., Hong W.K., Lotan R. (2000). Evidence that retinoic acid receptor beta induction by retinoids is important for tumor cell growth inhibition. J. Biol. Chem..

[37] Bai H., Li H., Li W., Gui T., Yang J., Cao D., Shen K. (2015). The PI3K/AKT/mTOR pathway is a potential predictor of distinct invasive and migratory capacities in human ovarian cancer cell lines. Oncotarget.

[38] Shin H.Y., Yang W., Lee E.J., Han G.H., Cho H., Chay D.B., Kim J.H. (2018). Establishment of five immortalized human ovarian surface epithelial cell lines via SV40 T antigen or HPV E6/E7 expression. PLoS ONE.

[39] Kim C.W., Oh E.T., Kim J.M., Park J.S., Lee D.H., Lee J.S., Kim K.K., Park H.J. (2018). Hypoxia-induced microRNA-590-5p promotes colorectal cancer progression by modulating matrix metalloproteinase activity. Cancer Lett..

[40] Therasse P., Arbuck S.G., Eisenhauer E.A., Wanders J., Kaplan R.S., Rubinstein L., Verweij J., Van Glabbeke M., Van Oosterom A.T., Christian M.C. (2000). New guidelines to evaluate the response to treatment in solid tumors. European Organization for Research and Treatment of Cancer, National Cancer Institute of the United States, National Cancer Institute of Canada. J. Natl. Cancer Inst..

