# Supplementary material for: ALDH1A2 Is a Candidate Tumor Suppressor Gene in Ovarian Cancer
Source: Cancers (Basel). 2019 Oct 14;11(10):1553. doi: 10.3390/cancers11101553 (PMC6826427; doi:10.3390/cancers11101553)
Supplement: Supplementary file 1 [file cancers-11-01553-s001.pdf]

Supplementary Materials

ALDH1A2 is a Candidate Tumor Suppressor Gene in Ovarian Cancer

Jung-A Choi, Hyunja Kwon, Hanbyoul Cho*, Joon-Yong Chung, Stephen M. Hewitt and Jae-Hoon Kim

Figure S1. Transfection efficiency after pCMV-tagB-Flag-ALDH1A2 transfection in RMG-I, SKOV3, and OVCA433 cells. Cells were grown on coverslips and transfected with pCMV-tagB-Flag-ALDH1A2 using Lipofectamine® 2000. Cells were then immunostained with primary antibodies for Flag and visualized by confocal microscopy (A). Transfection efficiency (%) was evaluated by counting cells expressing Flag.

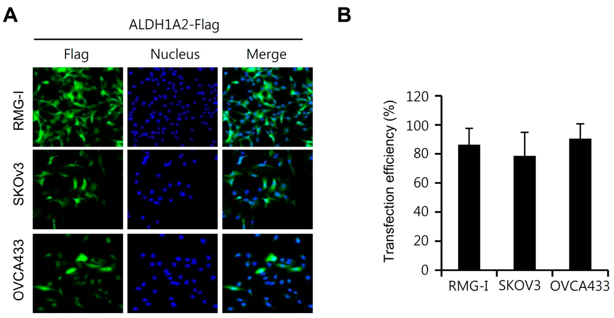

Figure S2. Effect of ALDH1A2 overexpression on cell cycle distribution and cell death in ovarian cancer. (A-B) Cells were transfected with pCMV-tagB-Flag-ALDH1A2 using Lipofectamine® 2000. After 24 h, the cells were harvested, fixed in ice-cold 70% ethanol, and stained with propidium iodide. Cell-cycle distribution (A) and cell death (B) were analyzed by fluorescence-activated cell sorting (FACS). Apoptotic cells were quantified for DNA content after propidium iodide staining; the sub-G_1 fraction (%) represents the proportion of apoptotic cells. Statistical significance was assessed using an unpaired t test. **$p < 0.05$, ***$p < 0.005$.

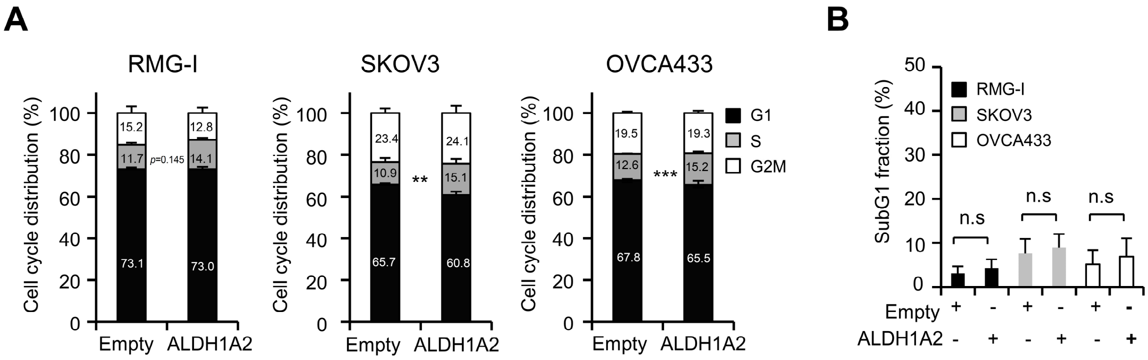

Figure S3. Annexin V/PI staining in RMG-I, SKOV3, and OVCA433 cells overexpressing ALDH1A2. (A-B) Cells were transfected with pCMV-tagB-Flag-ALDH1A2 using Lipofectamine® 2000. After the indicated time, cells were immediately stained with Annexin V-FITC and PI, subjected to flow cytometry analyses (A). Quantitative results of cell death were determined using Annexin V/PI (B).

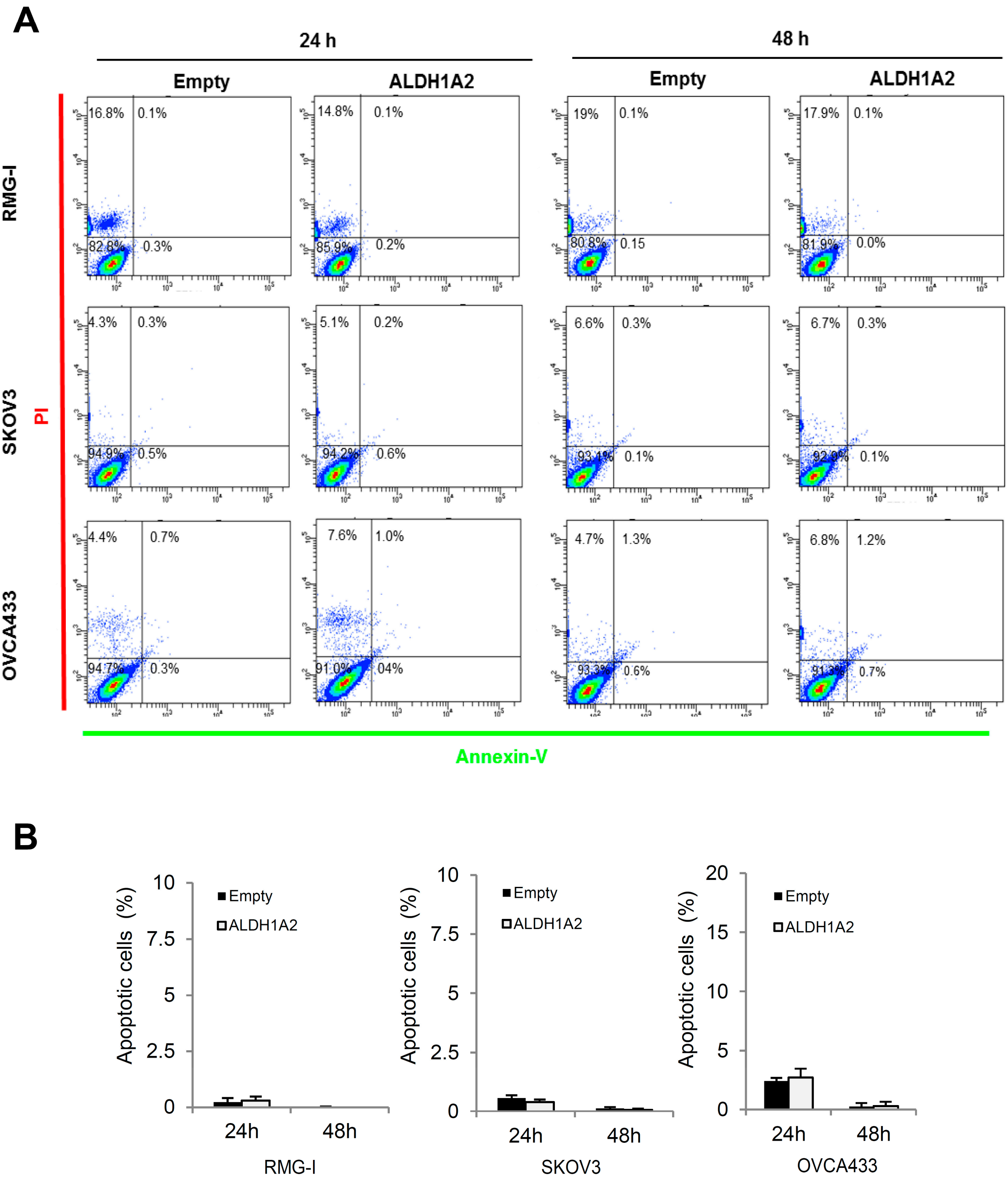

Figure S4. Methylation status of *ALDH1A2* genes in public datasets, Methylation and Expression Database of Normal and Tumor Tissues (MENT; http://mgrc.kribb.re.kr:8080/MENT/).

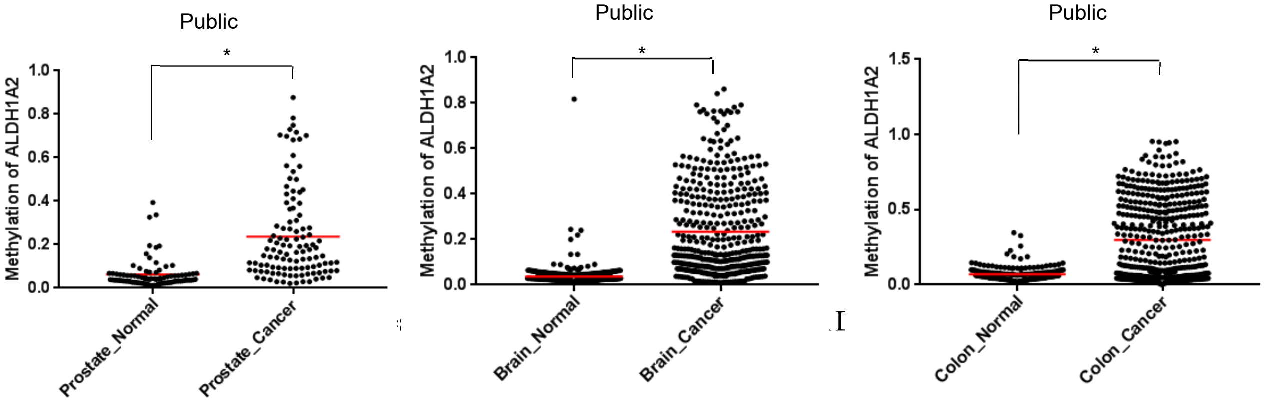

Figure S5. Expression of retinoic acid-related genes in ovarian cancer cell lines**.** Up- and downregulation of gene expression were examined in six ovarian cancer cell lines (YDOV-139, YDOV-157, YDOV-161, YDOV-13, YDOV-105, and YDOV-151) and four HOSE cell lines (HOSE 198, 209, 211, and 213) using DNA microarray. The values indicate fold-changes in the expression levels of each gene in the ovarian cancer cell lines relative to those in the HOSE cell lines. Data are expressed as the means ± SD. Statistical significance was assessed using an unpaired t test. **p* < 0.001, ****p* < 0.005.

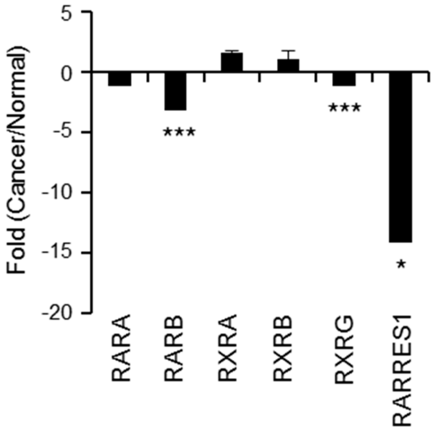

Figure S6. Data on the mRNA expression of *RARβ* and *RARRES1* obtained from the Oncomine database (http://www.oncomine.com). The Mann–Whitney test was used to evaluate statistical significances.

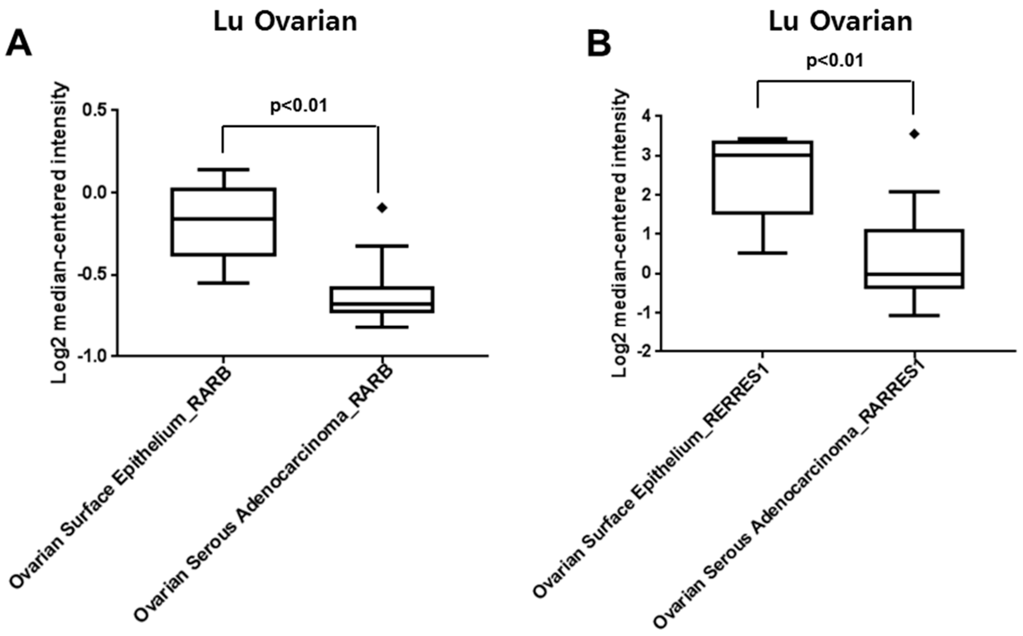

Figure S7. Original blots for western blot analyses shown in Figure 1D: (A) ALDH1A2, (B) β-actin.

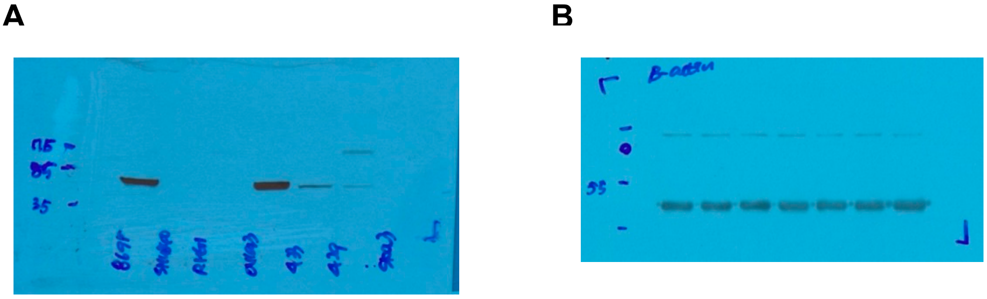

Figure S8. Original blots for western blot analyses shown in Figure 4A: (A)RMG1, Flag, (B) RMG1, ALDH1A2,(C) RMG1, β-actin, (D) SKOV3, Flag, (E) SKOV3, ALDH1A2, (F) SKOV3, β-actin, (G) OCAR433, Flag, (H) OVCA433, ALDH1A2, (I) OVCA433, β-actin.

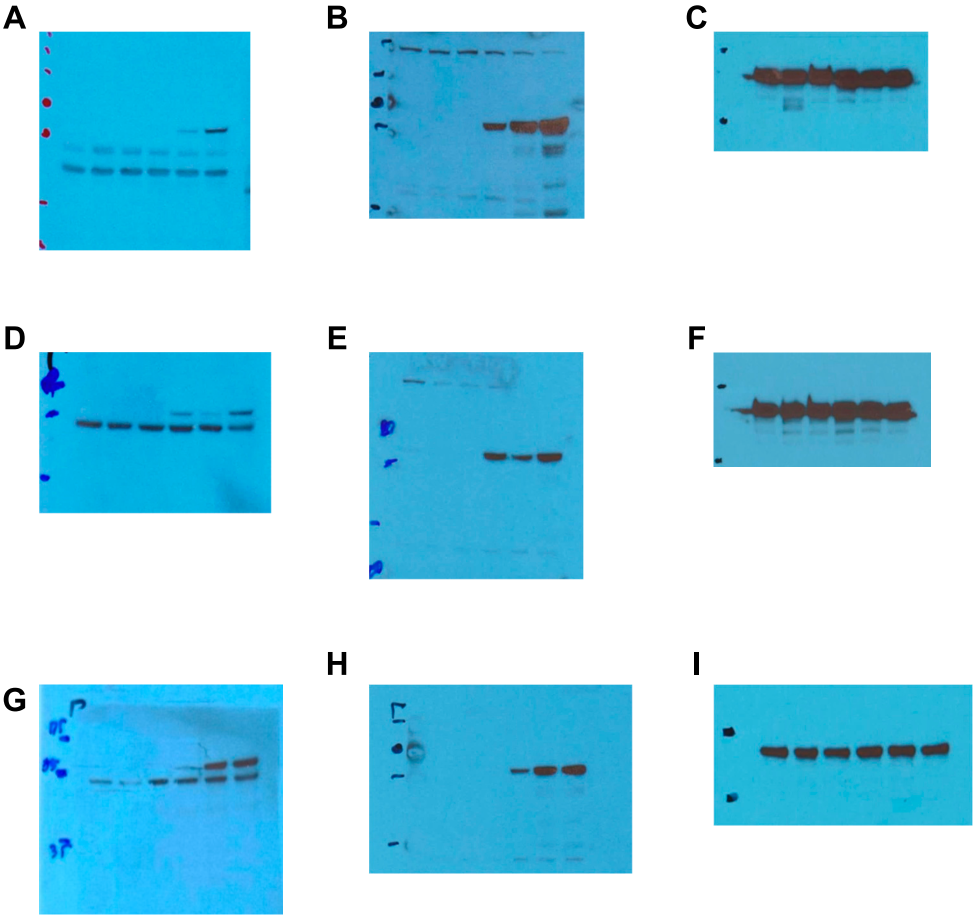

Table S1. Up- and downregulated *ALDH* genes in ovarian cancer cells

Symbol	Accession No.	Fold (Cancer/Normal)	SD (Cancer/Normal)	*p*-Value (Cancer/Normal)
ALDH1A1	NM_000689	1.73	2.57	NS
ALDH1A2	NM_170696	−52.78	0.82	<0.001
ALDH1A3	NM_000693	−5.96	2.71	NS
ALDH1B1	NM_000692	−5.17	1.72	<0.05
ALDH2	NM_000690	−1.26	0.74	NS
ALDH3B1	NM_001030010	−2.30	1.43	NS
ALDH3B2	NM_000691	2.01	1.01	NS
ALDH3A1	NM_000695	4.91	1.89	<0.05
ALDH3A2	NM_001031806	−2.13	1.36	NS
ALDH4A1	NM_003748	−1.26	0.70	NS
ALDH5A1	NM_001080	−1.14	0.88	NS
ALDH6A1	NM_005589	−1.35	0.72	NS
ALDH7A1	NM_001182	1.36	0.72	NS
ALDH9A1	NM_000696	−2.15	0.42	<0.001
ALDH16A1	NM_153329	1.24	0.72	NS

Up- and downregulation of the genes was examined in six ovarian cancer cell lines (YDOV-139, YDOV-157, YDOV-161, YDOV-13, YDOV-105, and YDOV-151) and four human ovarian surface epithelial (HOSE) cell lines (HOSE 198, 209, 211, and 213). The values indicate the fold-change of gene expression in the ovarian cancer cell lines relative to that in the HOSE cell lines. NS = not significant.

Table S2. Primer sequences for qRT-PCR and real-time PCR

Assay	Gene	Accession No.	Sequence (5′ to 3′)	Size (bp)
qRT-PCR	*ALDH1A2*	NM_003888.4	F, 5′- GATGCTGACTTGGACTATGCTGT-3′	210
			R, 5′-CTGTTTCTTATCAATCTGGGGAC-3′	
	GAPDH	NM_002046.7	F, 5′-GATCTCGCTCCTGGAAGAT-3′	146
			R, 5′-CAATGACCCCTTCATTGACC-*3′*	
Real-time PCR	*DNMT1*	NM_001130823	Forward, 5′- CCATCAGGCATTCTACCA-3′	132
			Reverse, 5′-CGTTCTCCTTGTCTTCTCT-3′	
	DNMT3A	NM_175629.2	Forward, 5′-CAATGACCTCTCCATCGTCAAC-3′	89
			Reverse, 5′-CATGCAGGAGGCGGTAGAA-3′	
	DNMT3B	NM_006892.4	Forward, 5′- CCATGAAGGTTGGCGACAA-3′	69
			Reverse, 5′-TGGCATCAATCATCACTGGATT-3′	
	ALDH1A2	NM_003888.4	Forward, 5′-GATGCTGACTTGGACTATGCTGT-3′	210
			Reverse, 5′ CTGTTTCTTATCAATCTGGGGAC-3′	
	18S rRNA	NM_022551.3	Forward, 5′-TCCAGGTCTTCACGGAGCTTGTT-3′	72
			Reverse, 5′-GGATGTAAAGGATGGAAAATACA-3′	

Table S3. Primer sequences for methylation-specific PCR

Gene	Sequence (5′ to 3′)
ALDH1a2-M	Forward, 5′- GTTTACGGTTAAGTTCGTTCGGCGTTTTC-3′
	Reverse, 5′- AAAACCACGACCCTCGCTCGACTTCG-3′
ALDH1a2-U	Forward, 5′-GTGTTTATGGTTAAGTTTGTTTGGTGTTTTT-3′
	Reverse, 5′- AAAACCACAACCCTCACTCAACTTCA-3′

M, methylated; U, unmethylated

Supplementary Materials and Methods

Public databases

DNA methylation analysis of the *ALDH1A2* gene in normal and ovarian tumor tissues of ovarian cancer patients was performed using the Methylation and Expression database of Normal and Tumor tissues (MENT; http://mgrc.kribb.re.kr:8080/MENT/), together with clinical data from the Gene Expression Omnibus (GEO) database (GEO accession numbers: for prostate cancer, GSE26126, GSE34174; for brain cancer, SE15745,GSE34356, GSE22891, GSE22867, and GSE34355; for colon cancer, GSE17648, GSE25062, GSE27130, GSE29490, and GSE36534) and The Cancer Genome Atlas (TCGA; for brain cancer, gbm dataset; for colon cancer, coad dataset). Methylation values were calculated based on the average beta value to measure methylation levels at each CpG-site, which range dfrom 0 (least methylated) to 1 (most methylated). No further normalization of derived beta-value was used.

Immunofluorescence

Cells were grown on coverslips, fixed in 4% paraformaldehyde for 30 min, and permeabilized with 0.2 % Triton™ X-100 for 10 min. Cells were washed twice with PBS, blocked with 3% bovine serum albumin (BSA), and incubated with anti-Flag antibodies (1:500; Santa Cruz Biotechnology, Dallas, TX, USA) at room temperature for 1 h 30 min. Cells were then incubated with Alexa Fluor® 488-conjugated goat anti-rabbit IgG at room temperature. DNA was stained with 100 ng/mL of 4′-6-diamidino-2-phenylindole (DAPI; Sigma-Aldrich, St. Louis, MO, USA). Slides were mounted using Prolong Gold anti-fade reagent (Life Technologies/Thermo Fisher Scientific, Carlsbad, CA, USA). Stained cells were viewed under an LSM710 confocal microscope with Zen software (Carl Zeiss, Jena, Germany). Transfection efficiency (%) was determined by counting the number of positive cells.

Cell growth assay

Cells were plated at a density of 2×10^{5} cells per well in 6-well plates. After 24 h, cells were transfected with pCMV-tagB-Flag-ALDH1A2 or an empty vector. After the indicated duration, cells were trypsinized at each time point and counted using the trypan blue exclusion method.

Annexin-V staining

Cell death was evaluated using Annexin V-FITC and propidium iodide (PI)-staining kit (Thermo Fisher Scientific) following the manufacturer's instructions. Briefly, 2×10^{6} cells were seeded in 100-mm dishes and transfected with pCMV-tagB-Flag-ALDH1A2 or an empty vector. After the indicated time, the cells were treated with trypsin and collected following washing with cold PBS. Then, 200 µL

binding buffer (Thermo Fisher Scientific) was used to resuspend the cells. Cells were stained with Annexin V-FITC and PI and further incubated for 5 min at room temperature. Stained cells were immediately subjected to flow cytometry analyses using FACS Canto II flow cytometer (BD Biosciences).
